# Poster Session II - A198 A SYSTEMATIC REVIEW: ROLE OF PYLORIC DILATION IN THE MANAGEMENT OF GASTROPARESIS

**DOI:** 10.1093/jcag/gwaf042.198

**Published:** 2026-02-13

**Authors:** M Gupta, A Barchi, A J Bredenoord, G J Masclee

**Affiliations:** Medicine, University of Calgary, Calgary, AB, Canada; Istituto di Ricovero e Cura a Carattere Scientifico San Raffaele Pisana, Milan, Lazio, Italy; Amsterdam UMC Locatie AMC, Amsterdam, NH, Netherlands; Amsterdam UMC Locatie AMC, Amsterdam, NH, Netherlands

## Abstract

**Background:**

Gastroparesis (GP) is a motility disorder characterized by delayed gastric emptying in the absence of obstruction. It is often associated with debilitating symptoms and has limited treatment options. Endoscopic pyloric-directed interventions, comprised of balloon or pneumatic dilations, aim to improve gastric outflow and alleviate symptoms. However, efficacy, safety and durability of pyloric dilation (PyD) in GP is uncertain.

**Aims:**

This systematic review evaluated clinical outcomes, safety, and durability of PyD in adults and children with non-surgical GP.

**Methods:**

A search of PubMed, Embase, and Scopus was conducted through March 2024 and identified studies (including abstracts) reporting outcomes of endoscopic PyD for GP. The review followed the Preferred Reporting Items for Systematic reviews and Meta-Analyses (PRISMA) checklist. Sixteen studies (12 full manuscripts, 4 abstracts) encompassing pediatric and adult populations met inclusion criteria. National Institute of Health Quality Assessment tool was used to evaluate risk of bias for the included studies

**Results:**

Over 700 patients from 1998 to 2023 were treated with PyD. Data extracted include patient demographics, GP diagnostic criteria, gastric emptying study (GES), Patient Reported Outcomes (PROs), dilation technique (balloon or pneumatic), follow-up duration, outcomes (GES and/or PRO change), and adverse events. Symptom improvement with PyD was reported in approximately 60–75% of patients, with durability ranging from several months to over one year. Pediatric studies demonstrated short-term efficacy with favorable tolerance. Repeated dilations were required in a subset, particularly in idiopathic and diabetic GP. Adverse events were limited to transient pain and/or minor bleeding. Heterogeneity in dilation protocols, outcome measures, and follow-up intervals prevented us performing pooled analysis.

**Conclusions:**

PyD appears to be a low-risk, safe option in GP. Despite variable follow up, most studies in this review demonstrated improvement in PRO and/or GES, even when PyD was combined with adjunctive therapies like Botox. Variability in PyD technique, heterogeneity in study design and non-standardized outcomes hinder assessment of long-term efficacy. Prospective controlled trials are needed, ideally those integrating physiologic metrics, such as EndoFLIP, to improve patient selection and identify those best suited for pyloric-targeted therapy

**Funding Agencies:**

None

